# Cholesteatoma of the external ear canal: etiological factors, symptoms and clinical findings in a series of 48 cases

**DOI:** 10.1186/1472-6815-6-16

**Published:** 2006-12-23

**Authors:** Hanne H Owen, Jørn Rosborg, Michael Gaihede

**Affiliations:** 1 Department of Otolaryngology, Head and Neck Surgery, Aalborg Hospital, Aarhus University Hospital, DK-9000 Aalborg, Denmark; 2 Department of Otorhinolaryngology, Dronning Ingrids Hospital, Postbox 3333, 3900 Nuuk, Grønland

## Abstract

**Background:**

To evaluate symptoms, clinical findings, and etiological factors in external ear canal cholesteatoma (EECC).

**Method:**

Retrospective evaluation of clinical records of all consecutive patients with EECC in the period 1979 to 2005 in a tertiary referral centre. Main outcome measures were incidence rates, classification according to causes, symptoms, extensions in the ear canal including adjacent structures, and possible etiological factors.

**Results:**

Forty-five patients were identified with 48 EECC. Overall incidence rate was 0.30 cases per year per 100,000 inhabitants. Twenty-five cases were primary, while 23 cases were secondary: postoperative (n = 9), postinflammatory (n = 5), postirradiatory (n = 7), and posttraumatic (n = 2). Primary EECC showed a right/left ratio of 12/13 and presented with otalgia (n = 15), itching (n = 5), occlusion (n = 4), hearing loss (n = 3), fullness (n = 2), and otorrhea (n = 1). Similar symptoms were found in secondary EECC, but less pronounced. In total the temporomandibular joint was exposed in 11 cases, while the mastoid and middle ear was invaded in six and three cases, respectively. In one primary case the facial nerve was exposed and in a posttraumatic case the atticus and antrum were invaded. In primary EECC 48% of cases reported mechanical trauma.

**Conclusion:**

EECC is a rare condition with inconsistent and silent symptoms, whereas the extent of destruction may be pronounced. Otalgia was the predominant symptom and often related to extension into nearby structures. Whereas the aetiology of secondary EECC can be explained, the origin of primary EECC remains uncertain; smoking and minor trauma of the ear canal may predispose.

## Background

External ear canal cholesteatoma (EECC) is a rare condition with an estimated incidence of 1.2 per 1,000 new otological patients[1]. EECC presents itself by an accumulation of epithelial debris in the ear canal, and early reports on such manifestations have been made in 1850 by Toynbee [2] and later in 1893 by Scholefield [3]. While these cases have appeared as EECC, they may also have represented cases of keratosis obturans, which has similar characteristics. In fact, the two terms have previously been used interchangeably, but since treatment strategies are different, the distinction between the two conditions is important [4-6]. Present definitions have mainly been based on a review by Piepergerdes *et al*. in 1980 [4], and a histopathological study by Naiberg *et al*. in 1984 [5]. While these studies have clarified definitions to some extent, a number of common features also remain, and most recently, Persaud *et al*. [6] reviewed the literature in attempt to define clearer distinctions. Their only conclusion was that there are still no reliable consistent symptoms or clinical signs that can differentiate between the two conditions; however, the most useful finding confirming an EECC is focal osteonecrosis or sequestration of bone lacking an epithelial covering [6].

These properties characterising the EECC also explain the extension of bony erosion seen in some cases with subsequent invasion of the adjacent structures of the temporal bone, like the mastoid [1,4,5,7-14], middle ear cavity [10,14] and exposure of the temporomandibular joint [1,11], and in rare cases dehiscence of the facial nerve [4,7,8,10,14] and the labyrinth [15]. Involvement of the sigmoid sinus [12] and the dura of the tegmen can also be found [14,16].

Symptoms like otorrhea and pain are often reported, but many cases can be remarkably silent or even asymptomatic [9,13]. Hence, EECC may be an insidious entity concealing serious destruction with few or no symptoms.

Classifications of the EECC can be based on pathogenetic theories. One classification has been suggested by Tos: 1) primary EECC, 2) secondary EECC, and 3) cholesteatoma associated with congenital atresia of the ear canal [17]. Secondary EECC is related to a variety of conditions mainly postoperative, although factors like recurrent inflammation as well as postinflammatory and posttraumatic stenosis or atresia with ear canal obstruction also occur [17]. In addition, radiation therapy incorporating the ear canal can also lead to EECC [12,18,19]. Whereas these conditions to some extent can be explained by their causes of origin, the aetiology in primary EECC is unknown [17].

Due to the sparse occurrence of EECC many studies only report on a few typical cases. In fact, we have found a mere 7 reports including more than 5 cases [1,8,9,13,14,19,20]. Hence, larger series providing stronger evidence of possible aetiological factors, symptoms and clinical findings are needed in order to improve identification and distinctions from keratosis obturans. This encouraged us to report on a larger series of patients from our department and on this background to review the current literature.

## Methods

The clinical records of all patients diagnosed with EECC in our department from January 1st, 1979 to December 31st, 2005 were retrospectively reviewed with focus on etiological factors, symptoms, and clinical findings. Forty-five patients with 48 cases of EECC were identified. All cases were classified according to Tos [17], and we found no competing courses, where the classification could be doubted. Our department is the only tertiary referral centre covering a county of around 600,000 inhabitants, and during the study period approximately 6750 new patients were referred to our department for assessment and ear surgery.

Our definition of EECC was primarily clinical. All patients showed a focal disruption of the skin with curling of the edges and underlying localised osteitis with bony destruction creating a cavity. In here we found invasion and accumulation of keratinized debris and in many cases sequestration of the underlying bone. The diagnosis was further supported by histological examinations revealing epithelial disruption and accumulation of keratinized debris containing various degrees of sequestered bone.

Smaller cholesteatoma pearls occasionally encountered after ear surgery were not included.

## Results

We found 25 cases (52%) of primary EECC and 23 cases (48%) of secondary EECC. According to their causes, these were described as postoperative (9 = 19%), postinflammatory (5 = 10%), postirradiatory (7 = 15%) and posttraumatic (2 = 4%). No cases associated with atresia or stenosis of the ear canal were identified. The distribution of cases including age, gender and side have been depicted in Table 1. The overall incidence rate amounted to 0.30 cases per year per 100,000 inhabitants and 7.1 cases per 1000 new otological patients.

**Table 1:** Distribution of cases, age, gender, and side (N = 48)*

**Classification**	**N (%)**	**Age†**	**♀/♂**	**Right/Left**
Primary	25 (52)	57 (33 to 82)	13/12	12/13
Secondary	23 (48)	-	14/9	9/14
postoperative	9 (19)	39 (16 to 56)	5/4	4/5
postinflammatory	5 (10)	48 (17 to 63)	3/2	1/4
postirradiatory	7 (15)	56 (32 to 66)	5/2	3/4
posttraumatic	2 (4)	16 (15 to 17)	1/1	1/1

* Two patients with primary and one patient with postirradiatory EECC had bilateral disease

† mean (range)

### Primary EECC

This group included two patients with bilateral disease. The major symptoms have been depicted in Table 2. Of the four cases with occlusion three had a conductive hearing loss, which was relieved by removal of debris.

**Table 2:** Distribution of symptoms*

**Classification**	**Otalgia**	**Otorrhea**	**Occlusion**	**Hearing loss†**	**Fullness**	**Itching**
Primary (N = 25)	15 (60)	1 (4)	4 (16)	3 (12)	2 (8)	5 (20)
Secondary (N = 23)	3 (13)	5 (22)	2 (9)	2 (9)	3 (13)	5 (22)
- postoperative (N = 9)	0	1 (11)	1 (11)	1 (11)	0	1 (11)
- postinflammatory (N = 5)	0	1 (20)	0	0	0	1 (20)
- postirradiatory (N = 7)	3 (43)	2 (29)	1 (14)	1 (14)	3 (43)	3 (43)
- posttraumatic (N = 2)	0	1 (50)	0	0	0	0

* Numbers of cases (percentages were determined within each group or subgroup)

† Only hearing loss attributed to the EECC was included

Larger areas of EECC including both the anterior, inferior and posterior walls were found in 10 cases; two-wall involvements were found in 6 cases, while one-wall in 9 cases. The cumulated involvements of the various parts have been depicted in Table 3. Involvements of adjacent structures included exposure of the temporomandibular joint in six cases, invasion of the mastoid in three cases, and both areas in one case. Another case involved the mastoid, where the erosion exposed the chorda tympani including its junction with the facial nerve. Middle ear involvement was only found in one case.

**Table 3:** Distribution of locations*

	**Anterior**	**Inferior**	**Posterior**	**Superior**
Primary (N = 25)	19 (76)	17 (68)	15 (60)	0
Secondary (N = 23)	11 (48)	15 (65)	15 (65)	1 (4)
- postoperative (N = 9)	6 (67)	5 (56)	6 (67)	0
- postinflammatory (N = 5)	3 (60)	4 (80)	3 (60)	0
- postirradiatory (N = 7)	2 (29)	5 (71)	5 (71)	0
- posttraumatic (N = 2)	0	1 (50)	1 (50)	1 (50)

* Number of cases within each group or subgroup (percentage of cases within each group or subgroup)

In 10 cases rigorous usage of Q-tips was reported. None of these cases revealed signs of inflammation. In addition, hearing aids or ear moulds for noise protection were used in six cases. In total either one or both of these mechanical factors were found in 12 of 25 cases (48%). In 12 cases the patients were smokers (48%), one case non-smoker, whereas no records were found in the remaining 12 cases.

### Postoperative EECC

Nine patients were found with EECC related to previous middle ear surgery (Table 1). Symptoms have been summarized in Table 2. One patient using hearing aid presented with a sensation of occlusion and hearing loss relieved upon removal of debris; otherwise symptomatic cases presented only intermittently and with one symptom in each case. In four cases a conductive hearing loss was reported attributed to previous middle ear disease.

The lesions were generally found smaller than in the group of primary EECC with only one case showing exposure of the temporomandibular joint. In three cases anterior, inferior as well as posterior involvement were found, two-walls in two cases, and one-wall in four cases. Table 3 summarizes the cumulated involvements.

The time from surgery until detection of the EECC was on average 7 years (range 1 to 22). Surgical procedures included simple myringoplasty (n = 3), tympanoplasty (n = 3), tympanoplasty with mastoidectomy (n = 3), in which one case included a radical cavity. The three cases of myringoplasty represented traumatic perforations caused by a blast injury, i.e. there was no history of other lesions than rupture of the tympanic membrane. The remaining indications for surgery were chronic otitis (n = 4) and cholesteatoma (n = 2). Four patients were smokers, three non-smokers and in 2 cases we have no records. Two patients used hearing aids. No use of Q-tips was reported.

### Postinflammatory EECC

Five patients presented with a history of infectious ear diseases (Table 1). Symptoms were few, since only one patient suffered from otorrhea and one from itching (Table 2). In three cases the lesions included the anterior wall exposing the temporomandibular joint. Two of these cases also included the inferior and posterior part. Two-wall involvement was found in one case, while one-wall in two cases. Table 3 depicts the cumulated involvements.

In two cases inflammation of the ear canal was related to previous recurrent otorrhea due to chronic otitis media with an eardrum perforation. One case had an active discharge at the time of EECC diagnosis, while the other had been dry for one year. The three remaining cases of which one used Q-tips had previous recurrent external otitis. Three patients were smokers, one non-smoker, while the last showed no record.

### Radiotherapy related to EECC

In six patients EECC developed after radiation therapy; one patient had bilateral affections (Table 1). Symptoms were more frequent in this group with three cases reporting otalgia, two otorrhea, three fullness, and three itching (Table 2). Apart from one case affecting both the anterior, inferior and posterior part, they were all minor affecting only one (n = 3) or two walls (n = 3) (Table 3). In one case a tympanic membrane perforation was found and cholesteatoma debris invaded the middle ear cavity.

The time from completion of radiation therapy to the recognition of the EECC was on average 5 years (range 3 to 12). One patient initially demonstrated two smaller EECC with bony erosion in the posterior wall 2 years after radiation. These were managed by cleansing, but 11/2 years later another EECC developed inferiorly with an extension into the middle ear that needed surgery. In this case healing was insufficient, and demanded repeated surgery. Three more case demanded surgery, while one case was managed by a smaller procedure under local anaesthesia. Two cases were managed by cleansing only. In five patients smoking was reported, while this information lacked in the last patient. One patient used hearing aids, one patient Q-tips, and one patient both.

### Posttraumatic EECC

This group consists of two patients both developing EECC 4 and 60 months, respectively, after severe head injuries with skull-base fractures. The first patient had no symptoms except for a 20–35 dB conductive hearing loss explained by an ossicular disruption. At surgery fracture lines at 1 and 7 o'clock were revealed; in the latter epithelium was growing into the fracture line presenting as a smaller EECC with no extensions into adjacent walls or structures. The other patient had persistent otorrhea and a 30–35 dB conductive hearing loss caused by erosion of the incus. At surgery, several fracture lines were found and EECC's were revealed in two of these, one superior and one posterior with extensions through the mastoid into the atticus, antrum and middle ear. No information was found on smoking or Q-tips.

## Discussion

### Incidence and distribution of types

The rare occurrence of EECC makes it difficult to establish incidence rates, and they have not been reported directly in the literature. From Anthony and Anthony an incidence of 1.2 primary cases per 1,000 new otological patients can be estimated [1]. This corresponds well to 1.7 per 1,000 patients estimated by Vrabec and Chaljub [21]. The incidence of primary cases calculated from our material was higher, 3.7 cases per 1,000 patients, whereas the incidence of all cases was 7.1 per 1,000 patients. However, this latter figure concurs well with Vrabec and Chaljub, who found a total incidence of 1 in 200, i.e. 5 cases per 1,000 patients [21].

The incidence rate from our study was 0.15 for primary cases, while 0.30 for all cases per year per 100,000 inhabitants; in comparison, the incidence rate of middle ear cholesteatoma is around 9.2 per year per 100,000 [22]. We have not found comparable data in the literature, since previous studies lack information on the background population. The population of our county has been fairly constant over the study period, and thus, the estimate seems reliable, also since the incidence related to the number of patients is comparable to previous reports [1,21].

Our department functions as the only tertiary referral centre in our county receiving patients primarily for surgery, but also assessments of rare conditions like EECC. In accordance with the histopathological classification suggested by Naim *et al. *[20], all our cases presented as stage III or IV with osteitis, localized invasion and bony destruction including extensions into adjacent structures in some cases. Hence, less severe cases with hyperplasia and periosteitis only have not been included in our material (stage I and II) [20], but probably treated conservatively by our private otological practises (secondary referral centres). This obviously influences the incidences, and some variations should be expected due to local differences of practice including referral patterns [21]. In addition we do not know how many cases presenting no or vague symptoms remain undiagnosed. The appearance of the cases occurred at random over time i.e. the incidence seemed to be constant during the study period.

The distribution of the EECC showed that 52% of cases were primary (Table 1). From the few previous reports, where similar distributions can be calculated, this proportion varies between 41 and 62% [13,14]. The second most common type was the postoperative group (19%), which also concurs with previous studies; the percentages from these amounted to 34 and 23%, respectively [13,14]. The remaining groups show larger differences between studies explained by the small numbers in each group, but also by differences in definitions. For instance, postinflammatory EECC was found less frequent by Vrabec and Chaljub (5%) [13] compared with our results (10%), which included cases of inflammation due to both recurrent external otitis as well as otitis media; Vrabec and Chaljub only reported cases with external otitis [13]. No cases of postirradiatory EECC were found by Vrabec and Chaljub [13] or Heilbrun *et al. *[14], whereas we found 15% related to radiotherapy. In contrast, we found no cases related to posttraumatic or postinflammatory stenosis or atresia of the ear canal predisposing for development of an EECC [15,17].

### Distributions of age, gender and side

While the data on secondary EECC reflects their causes of origin, the data describing the primary cases are more likely to characterize the EECC itself. Hence, the following section refers to primary cases only.

The age distribution of primary EECC showed a mean of 57 years (range 33 to 82; Table 1). Earlier studies found a higher mean age [4,9], but more recent studies[1,8,13] support our findings and confirm that EECC can also be found in younger patients.

The gender distribution of our group showed a female/male case ratio of 13/12 (Table 1). Anthony and Anthony found a case ratio of 7/5 [1], Sismanis *et al*. a ratio of 4/6 [8], and Holt reported a ratio of 2/6 [9]. Some studies only include smaller number of cases, but the cumulated data from these, including our current results, amounts to a ratio of 26/29, i.e. an overall ratio indicating a random risk of gender regarding primary EECC.

The side of affection showed a right/left case ratio of 12/13 (Table 1). Previously, both left-sided [1,8] and right-sided predominance [7,9] has been reported. The cumulated ratio from these studies including our data amounts to 26/31 suggesting a random occurrence. In two patients bilateral affections were found, i.e. a ratio of 2 in 23 patients. Similar ratio can be determined from Anthony and Anthony (2/10) [1], Sismanis *et al. *(2/8) [8], Holt (2/6) [9], and Vrabec and Chaljub (5/13) [13]. Thus, the ratio of bilateral disease based on these studies including our own results varies greatly (0.09 to 0.38), but the overall ratio amounted to 0.22 (13/60).

The numbers of cases in the groups of secondary EECC were all smaller, but to some extent they were characterized by the causes of their origin. For instance, the age distribution of postoperative cases showed a mean of 39 years (range 16 to 56), which is markedly lower than for primary EECC (Table 1). This obviously reflects the wider range of age of patients submitted for ear surgery, including children [9,13,15].

### Symptoms

The most common presenting symptoms reported in the literature are otalgia and otorrhea. Otalgia has been described as a more vague or mild discomfort [1,9], but also as a chronic dull pain [4], and in some cases severe pain [7]; thus, the symptom is not described consistently [6]. The incidence of major symptoms has been extracted from selected studies and compared with our results in Table 4; only data from primary cases are included, since they contain the more substantial number of cases.

**Table 4:** Distributions of symptoms in primary EECC for different studies*

	**Otalgia §**	**Otorrhea**	**Occlusion**	**Hearing loss**	**Fullness**	**Itching**	**Asymptomatic**
Owen *et al. *(2006)	15/25 (60)	1/25 (4)	4/25 (16)	3/25 (12)	2/25 (8)	5/25 (20)	6/25 (24)
Vrabec and Chaljub (2000)^13^†	1/13 (8)	7/13 (54)	-	0	-	1/13 (8)	4/13 (31)
Holt (1992)^9^	1/8 (13)	2/8 (25)	-	1/8 (13)	3/8 (38)	-	2/8 (25)
Sismanis *et al*. (1986)^8^†	3/8 (38)	3/8 (38)	-	2/8 (25)	1/8 (13)	1/8 (13)	-
Anthony and Anthony (1982)^1^	7/12 (58)	3/12 (25)	-	2/12 (17)	1/12 (8)	-	-
Piepergerdes *et al*. (1980)^4^	2/3 (67)	3/3 (100)	-	0	-	-	0
Smith and Falk (1978)^7^	2/2 (100)	-	-	-	-	-	-

* Number of cases with symptom/total number of EECC (percentage with symptom)

§ Otalgia refers to both pain and discomfort

† Number of patients with symptom/total number of patients (percentage with symptom). These groups consisted of more bilateral cases, but information on symptoms was only given for the number of patients

In 15 of 25 primary cases (60%), and only in 3 of 23 secondary cases (13%) we found otalgia reported (Table 2). Whereas this previously has been held as a major symptom [4,7], more recent studies have shown great variation with incidences between 8 and 58% (Table 4). This variation probably reflects problems defining otalgia, but it may also be related to the extension of the EECC. Hence, we found otalgia in the primary group in 6/7 cases with exposure of the temporomandibular joint, and in 3/4 cases with mastoid involvement. The extension within the ear canal itself seemed insignificant, since our 10 primary cases with 3 parts of the wall affected were divided evenly between otalgia and no otalgia (5/5). In conclusion, pain or discomfort is an inconstant symptom as pointed out by Persaud *et al*. [6], though it may be related to the extent of disease.

Otorrhea was found only in six cases in total (13%; Table 2), though this has also been reported as a frequent symptom [4,8,9,12,13]. In Table 4 the incidence of otorrhea in primary EECC from other studies varies between 25 and 100%, and thus, it can also not be held as a consistent symptom [6]. The number of ears with discharge was small, and we were unable to relate it to the extension of the EECC or coexisting otalgia.

Occlusion was found in 13% of the cases. We defined occlusion as the patient's subjective feeling of having the ear canal occluded often with a concurrent conductive hearing loss relieved upon removal of debris [1,8]. Five out of six patients with occlusion had conductive hearing loss, which was related to the accumulation of debris (Table 2). In primary cases hearing loss varies between 12 and 25% (Table 4), and thus, hearing loss is inconsistent and mostly seen, when the EECC occludes the ear canal [1,4,9].

Fullness is another more vaguely defined subjective feeling in the ear canal reported in some studies [1,8,9]. In the present context we defined it as a sensation in the ear that was neither otalgia nor occlusion, and found this reported in five cases (10%).

It is interesting to note that 24% of our primary cases were asymptomatic. Similar proportions of asymptomatic patients have previously been found between 25 and 31% (Table 4) [9,13]. Thus, a considerable number of the cases are found for other reasons, for instance postoperative checks or routine wax cleaning [9].

### Location and extension of the EECC

Our primary cases were all found in the anterior (76%), inferior (68%) and posterior wall (60%) of the ear canal, whereas none in the superior part (Table 3). Anterior and inferior location was emphasized by Anthony & Anthony [1], a posterior and inferior predominance by Heilbrun *et al*. [14], while Piepergerdes *et al*. described all these areas to be included [4]. Altogether this seems to reflect an even distribution of location between the anterior, inferior and posterior walls. In some cases also the superior wall can be involved [9,14], and even circumferential cases similar to keratosis obturans have been described [14].

In general, the secondary cases, apart from one posttraumatic case, were less extensive, which may also explain the less prominent symptoms in these groups compared with primary cases (Tables 2 and 3). The postoperative and postinflammatory cases exhibited almost even distributions between areas, whereas postirradiatory cases tended primarily to affect the inferior and posterior part of the ear canal. However, each group contained only few cases, and we have found no systematic data reported for comparison.

The invasion of adjacent structures only consisted of a minor fraction of the cases. Distribution of invasions has only been provided by Heilbrun *et al*., which has been included in Table 5 for comparison [14]. In general we found mastoid and middle ear involvements relatively frequently, but in addition a remarkably high proportion of our cases were found to present with exposure of the fibrous capsule of the temporomandibular joint. Especially, a large proportion of the postinflammatory cases (60%) showed temporomandibular joint exposure, whereas the primary group contained 7 cases resulting in a primary case ratio of 7/25 (28%). This has only been sparsely reported in the literature [1,11]. We have no explanation for the predominance of joint involvement in postinflammatory cases, as well as the high incidence in primary cases.

**Table 5:** Extensions of the EECC into adjacent structures*

	**TM joint§**	**Mastoid**	**Middle ear**	**Tegmen**	**NVII erosion**	**Atticus**	**Antrum**	**No extension**
Owen *et al*. 2006†	11 (23)	6 (13)	3 (6)	0	1 (2)	1 (2)	1 (2)	30 (63)
Heilbrun *et al*. 2003^14^#	0	4 (31)	5 (38)	1 (8)	2 (15)	-	-	-

* Number of cases (percentages)

† N = 48

# N = 13

§TM joint = temporomandibular joint

### Etiological factors

The primary or spontaneous EECC describes an apparently idiopathic form, where the pathogenetic events are unclear. It has been hypothesized that the EECC is a reactive process due to a primary underlying osteitis [1,4,5]. However, mechanical factors (Q-tips, hearing aids) resulting in primary inflammatory changes of the skin, as well as smoking resulting in tissue ischemia have also been suggested [19]. Alternately, age-related changes in epithelial migration and cerumen glands resulting in a drier wax composition have been considered factors leading to entrapment and accumulation of epithelial cells [9]. In partial support of this hypothesis, Makino and Amatsu have demonstrated slower migration rates in the inferior wall in patients with EECC, and similarly suggested that it could be explained by hypoxic conditions due to poor blood supply[23].

In 48% of our primary cases mechanical factors were plausible, while in the remaining cases no information was found. Smoking was reported in 48% of the cases, non-smoking in 4% while the rest were unreported. The secondary cases contained smaller numbers, but the overall number of smokers was 12, while four were non-smokers and in six cases no information was found. Thus, among those asked about their smoking habits there was an overall rate of 83% smokers (24/29). Q-tips were used in one postinflammatory case and two postirradiatory cases, hearing aids in two postoperative cases and two postirradiatory cases. Altogether, these figures seem rather inconclusive on the role of mechanical factors in the postinflammatory, postoperative and posttraumatic groups, whereas both a mechanical factor and smoking were found in 5/7 (71%) of the postirradiatory cases. This may suggest that fragile skin after radiation therapy is more likely to be affected from mechanical factors and hypoxia due to smoking.

In general we cannot conclude from these results whether use of Q-tips led to EECC, or whether the irritation from EECC led to Q-tip use.

The aetiology of secondary cases of EECC can be more easily explained. Thus, the postoperative cases have been explained by entrapment of keratinized epithelium under the graft or skin flap [8,13,16,17]. We found nine postoperative cases of EECC in approximately 6750 unselected otological patients, i.e. a risk of 1.3 per 1,000 surgeries representing a variety of procedures. In comparison a risk of one EECC in 3,000 stapedectomies has been reported [1]. The higher risk found in our group can be explained by the wider range of surgical procedures, including grafting of the tympanic membrane, which is not performed in stapes surgery. The latency between primary surgery and detection of the EECC was on average 7 years (range 1 to 22); a similar average of 6 years (range 0.3 to 16)) has been reported by Vrabec and Chaljub [13]. Thus, the latency period can be long, and a larger part of these patients may not be diagnosed during the regular postoperative checks.

In posttraumatic EECC a similar entrapment of epithelium in fracture lines or bony defects can occur in addition to accumulation due to posttraumatic stenosis of the ear canal [13,15,17]. The two cases found in our series showed a latency of 1 to 5 years. In agreement, Brookes and Graham reported latencies between 6 months and 4 years in three patients [15].

The postinflammatory EECC is mostly associated with atresia or stenosis of the ear canal [13,17,21], whereas EECC found after chronic or recurrent discharge based on otitis media, as found in two of our patients, has been more rarely reported [11,19]. These cases suggest that osteitis and invasion of epithelium were secondary to inflammatory changes of the skin.

Postirradiatory EECC is sparsely reported including only a few separate cases [12,18,19]. Radiation therapy covering the area of the external ear canal leads to both soft tissue changes including epithelial hyperplasia and subsequently atrophic changes as well as osteitis including necrosis [18]. Thus, the primary pathogenetic events are also not clear in these cases. We found that latencies between therapy and discovery of the cholesteatoma were on average 5 years (range 3 to 12). Martin *et al*. reported one case 12 years after therapy [12], while Farrior found one case after 20 years [19]. One particular problem emerged in this group, namely the insufficient healing after surgery in one of the cases making re-operation necessary. This problem can be expected due to decreased tissue viability in response to previous radiation.

More recently, immunohistochemical investigations have been introduced, reporting increased levels of various growth factors in EECC specimens [24]. These also include elevated vascular endothelial growth factor indicating tissue hypoxia [25], as well as increased hepatocyte growth factor involved in an increased apoptosis of epithelial cells and debris formation [26]. This line of investigations may add valuable basic information on the aetiology.

### EECC versus keratosis obturans

The more classical distinction between EECC and keratosis has been based on Piepergerdes *et al *[4], and later addressed by Persaud *et al*. [6]. However, more of the classical experiences are not in accordance with our findings. Hence, the presence of both dull pain and otorrhea in EECC has been underlined [4], whereas we only found these symptoms in 60 and 4% of the primary cases (Table 2). Further, the age distribution in EECC is not limited to older patients [4], since we found a mean age of 57 years (range 33 to 82), and the appearance is not limited to unilateral affections [4], since all together bilateral cases seem to constitute 20% of the patients.

However, agreement was found in as much as in EECC hearing loss was infrequent, and pain was more likely characterised by a dull pain and never as an acute severe pain found in keratosis obturans [4]. Moreover, we also found that the lesions were localised as well as the tympanic membrane was generally normal in contrast to the more general affection of keratosis obturans with inflammation of the ear canal skin and the tympanic membrane [4,6]. In addition, we found no lesions in the superior part of the ear canal apart from one case, where it was explained by the ingrowth into a fracture line (Table 3).

### Treatment strategies

The mean follow up period was 2 years (0.25 to 12) and only two recurrences were found during follow up: one primary case (1/25 = 4%) and one postirradiatory case (1/7 = 14%). Most authors have found that small lesions can be treated conservatively or by smaller procedures under local anaesthesia, whereas larger lesions need proper surgery removing the cholesteatoma, burring off affected bone areas, and grafting defects with fascia [1,8,9,13,14,19]. However, even small lesions may represent osteitis resistant to conservative treatment [11], and a more aggressive attitude favouring surgical approach can be argued for [1,4,5,11,15]. This more radical approach was applied in our series. An enaural approach was used in 22 cases and postauricular in 22 cases. All affected skin was removed to leave surrounding healthy skin edges and the meatal skin was raised. The bone was drilled off to leave no residual disease. Generous grafting with fascia was applied followed by packing for three weeks. In five extensive cases cartilage was used to rebuild the ear canal. Conservative treatment may be favoured in postirradiatory cases due the impaired healing, but generally the treatment was successful, and hence, we have not focused on analysing these aspects.

## Conclusion

EECC is a rare condition with an estimated total incidence rate of 0.30 cases per year per 100,000 inhabitants. Primary EECC affects a wide range of ages including younger adults. The affections are most often located in the anterior, inferior, and posterior parts of the ear canal with exposure of temporomandibular joint as the most common involvement of adjacent structures. Symptoms including otalgia and otorrhea are often vague or inconsistent. Diagnosis is therefore highly reliant on its recognition at clinical examination by the ENT specialist. Focal skin disruption, osteonecrosis, and varying sequestration should favour a diagnosis of EECC as opposed to keratosis obturans. Mechanical trauma and smoking may be predisposing factors.

## Competing interests

The author(s) declare that they have no competing interests.

## Authors' contributions

HHO took part in the design of the study, acquisition, analysis and interpretation of data, and drafting of the manuscript. JR conceived of the study, participated in the acquisition of data, the analysis and interpretation of data and revised the manuscript critically for important intellectual content. MG took part in acquisition, analysis and interpretation of data, and drafted the manuscript. All authors have read and approved the final version of the manuscript.

## Pre-publication history

The pre-publication history for this paper can be accessed here:


